# Proteomic analysis reveals novel ligands and substrates for LNX1 E3 ubiquitin ligase

**DOI:** 10.1371/journal.pone.0187352

**Published:** 2017-11-09

**Authors:** Joan A. Lenihan, Orthis Saha, Paul W. Young

**Affiliations:** School of Biochemistry and Cell Biology, University College Cork, Cork, Ireland; Augusta University, UNITED STATES

## Abstract

Ligand of Numb protein X1 (LNX1) is an E3 ubiquitin ligase that contains a catalytic RING (Really Interesting New Gene) domain and four PDZ (PSD-95, DlgA, ZO-1) domains. LNX1 can ubiquitinate Numb, as well as a number of other ligands. However, the physiological relevance of these interactions *in vivo* remain unclear. To gain functional insights into the LNX family, we have characterised the LNX1 interactome using affinity purification and mass spectrometry. This approach identified a large number of novel LNX1-interacting proteins, as well as confirming known interactions with NUMB and ERC2. Many of the novel interactions mapped to the LNX PDZ domains, particularly PDZ2, and many showed specificity for LNX1 over the closely related LNX2. We show that PPFIA1 (liprin-α1), KLHL11, KIF7 and ERC2 are substrates for ubiquitination by LNX1. LNX1 ubiquitination of liprin-α1 is dependent on a PDZ binding motif containing a carboxyl terminal cysteine that binds LNX1 PDZ2. Surprisingly, the neuronally-expressed LNX1p70 isoform, that lacks the RING domain, was found to promote ubiquitination of PPFIA1 and KLHL11, albeit to a lesser extent than the longer RING-containing LNX1p80 isoform. Of several E3-ligases identified in the LNX1 interactome we confirm interactions of LNX1 with MID2/TRIM1 and TRIM27. On this basis we propose a model whereby LNX1p70, despite lacking a catalytic RING domain, may function as a scaffold to promote ubiquitination of its ligands through recruitment of other E3-ligases. These findings provide functional insights into the LNX protein family, particularly the neuronal LNX1p70 isoform.

## Introduction

**Fig 1:**
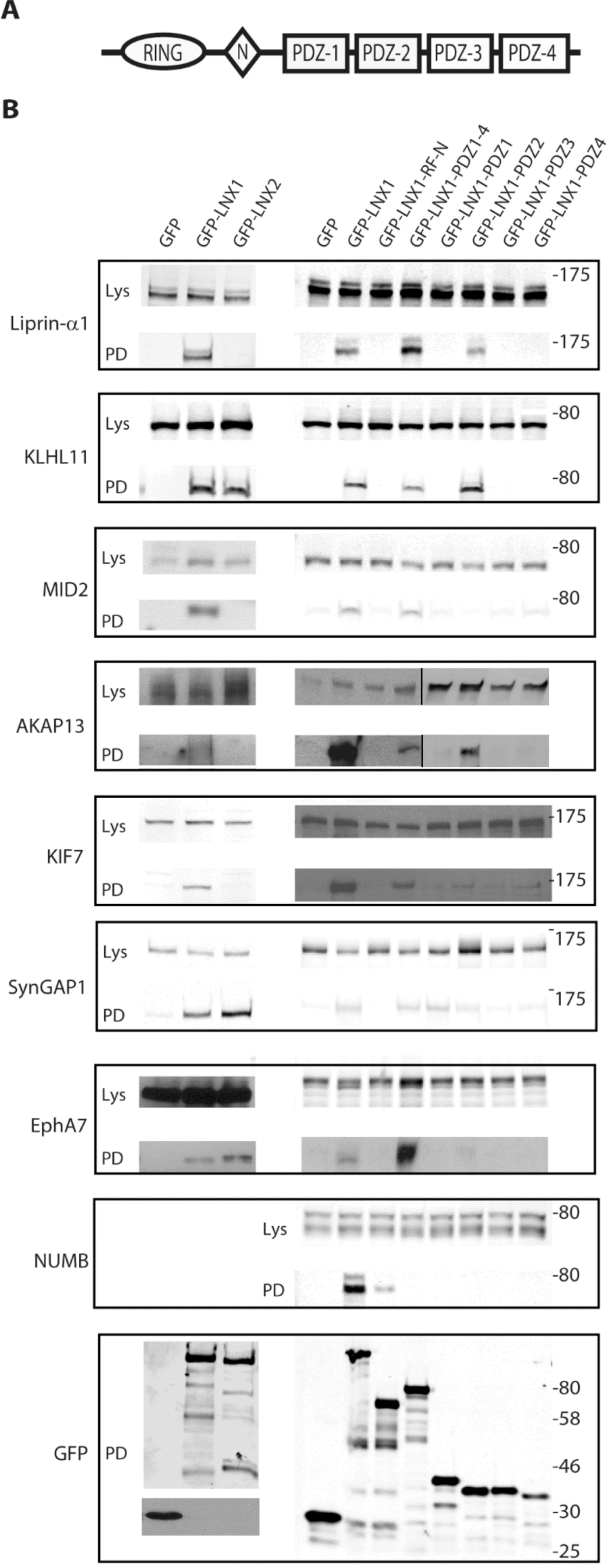
Characterisation of LNX1 interacting proteins. **(A)** Schematic diagram of the domain structure of LNX1p80 and LNX2 showing the RING and four PDZ domains. N represents the NUMB-binding NPAY/NPAF motif. **(B)** The ability of the indicated proteins to interact with transfected GFP-tagged LNX constructs was assessed in HEK 293 cells. For each interacting protein, top panels show western blots of cell lysates (Lys), while the bottom panels show the output of a GFP “pull down” assay (PD). In the panels on the left, the specificity of interactions for LNX1 versus LNX2 was assessed, while on the right the interaction site on LNX1 was mapped to individual protein domains. Binding of endogenous proteins to LNX was assessed for liprinα-1, KIF7 and NUMB. For the other proteins, interactions of transfected HA or GST epitope-tagged proteins were assessed. For AKAP13, the mapping to LNX domains was performed in two separate experiments. Successful expression and pull down of GFP-tagged LNX proteins was verified in all assays and representative “pull down” blots probed for GFP are shown. n = 2–3.

Ligand of Numb protein X1 (LNX1) was first characterised based on its ability to bind to the cell fate determinant protein, NUMB [1]. This ability is shared by the closely related LNX2 protein [2]. LNX1 and LNX2 have the same domain structure, comprising an amino-terminal RING (Really Interesting New Gene) domain, a NUMB-binding motif (NPAY or NPAF) and four carboxyl-terminal PDZ (PSD-95, DlgA, ZO-1) domains (Fig 1A). Three major isoforms of LNX1 have been described; the non-neuronal LNX1p80 isoform and two shorter, brain-specific, isoforms, LNX1p70 and LNX1p62, that lack the RING domain but contain the NPAY motif. LNX1p80, through its RING domain, can ubiquitinate specific isoforms of NUMB, thereby targeting NUMB for proteasomal degradation [3, 4]. While *Lnx*1 and *Lnx2* mRNAs are widely expressed in several adult tissues, the earliest embryonic expression of both genes is observed in the central nervous system (CNS) [1, 2, 5]. Two recent studies have proposed a role for LNX2 in modulating neurogenesis in the sub-ventricular zone of the developing brain [6, 7]. In agreement with a role for LNX proteins in regulating neural development, double knockout mice lacking both LNX1 and LNX2 in the central nervous system exhibit reduced anxiety-related behaviour [8]. The molecular basis for this phenotype is unclear however, and notably no alterations in NUMB levels were seen in these mice. Other studies suggest roles for LNX proteins in cancer, immune function and bone homeostasis [9–11], and in zebra fish LNX2 is important for exocrine cell differentiation in the pancreas [12]. However our understanding of such putative functions of mammalian LNX1 and LNX2 proteins *in vivo* is very incomplete.

The combination of a RING and one or more PDZ domains is a unique feature of the LNX family [13]. PDZ domains function as protein-protein interaction modules, most commonly binding to the carboxyl-termini of other proteins. Wolting *et al*. [14] compiled a list of 220 LNX-interacting proteins both from their own work and the published literature, while a subsequent study by Guo *et al*. [15] identified a further 30 LNX-interacting proteins. Most studies of LNX interacting proteins to date have employed either yeast-two hybrid assays or arrays of PDZ domains and PDZ-binding motifs. The vast majority of interactions identified involve the LNX PDZ domains. Apart from NUMB [4], only a small number of the other described LNX-interacting proteins have been shown to be substrates for ubiquitination by LNX. For example, overexpression of LNX1 was shown to cause ubiquitination and proteasomal degradation of c-Src and PBK (PDZ binding kinase) [15, 16], while LNX-mediated ubiquitination of claudins and CD-8α triggers endocytosis of these transmembrane proteins from the cell surface [10, 17]. These examples demonstrate that the ubiquitin ligase activity of LNX RING domains can be directed towards specific substrates via PDZ-mediated interactions. Given the low and potentially cell-type restricted expression patterns of LNX proteins [16, 18], it is important to identify physiologically relevant interacting proteins and substrates in order to elucidate the *in vivo* functions of mammalian LNX proteins.

To gain further insights into the poorly understood LNX proteins, we sought to examine the molecular interactions of the full length LNX1 protein in mammalian cells under conditions that are more physiologically relevant than previous studies. To that end, we have characterised the LNX1 interactome using affinity purification and mass spectrometry. Our results validate some known LNX interactions and identify a significant number of new ones. We show that several of these novel ligands co-localize with LNX1 in cultured cells and are substrates for ubiquitination by LNX1. In addition, we provide evidence that the neuronal LNX1p70 isoform that lacks the RING domain, may nevertheless promote ubiquitination of interacting proteins possibly through recruitment of other E3 ligases into multi-protein complexes. This proteomic analysis thus provides novel insights into LNX1 protein function.

## Materials and methods

### Antibodies and cDNA constructs

The coding sequences of mouse *Lnx1* (p80 isoform) and *Lnx2* were cloned into the pEGFP-C2 vector (Clontech). Empty pEGFP-C2 vector was used to express EGFP alone. LNX1 sequences in pEGFP-C2, for mapping interactions, encoded the following amino acids (aa) based on the LNX1p80 protein sequence: RING-NPAY motif, aa1-277; PDZ1-4, aa268-728; PDZ1, aa271-384; PDZ2, aa377-470; PDZ3, aa500-598; PDZ4, aa630-725. MID2 constructs based on transcript variant 2 of human MID2 (NM_052817) encoded the following amino acids: full length MID2, aa 1–705; RING domain—BBOX type Zinc finger-2, aa 1–235; Coiled coil–SPRY domain, aa 222–705; Coiled coil-COS domain, aa 222–406; FN3 domain—SPRY domain, aa 395–705. MID1 and TRIM 27 constructs encoded aa 1–667 of human MID1 (NM_000381) and aa 1–513 of human TRIM27 (NM_006510) respectively. Coding sequences for LNX interacting proteins were cloned into the vectors pCMV-N-HA, pCMV-N-FLAG or pDEST27 to produce proteins with amino-terminal HA, FLAG and GST tags respectively. The following antibodies were used: anti-GFP (Green Fluorescent Protein, catalogue number ab290, Abcam), anti-HA (catalogue number MMS-101R, Covance), anti-GST (Glutathione-S-Transferase, catalogue number G1160, Sigma-Aldrich), anti-FLAG (catalogue number F3165, Sigma-Aldrich), anti-liprin-α1 (catalogue number ab26192, Abcam), anti-liprin-α3 (catalogue number 169102, Synaptic Systems), anti-KIF7 (catalogue number ab95854, Abcam), anti-NUMB (catalogue number NB500-178, Novus Biologicals), anti-AKAP13 (catalogue number NB100-68214, Novus Biologicals). Horse radish peroxidase, Dylight 488 and Cy3 conjugated secondary antibodies were purchased from Jackson ImmunoResearch, while IR_700_ and IR_800_ conjugated secondary antibodies were from Li-Cor Biosciences. All other reagents were from Sigma-Aldrich unless stated otherwise.

### Purification of LNX1 complexes from stably-transfected cells

HEK (Human Embryonic Kidney) 293 cells (ATTC), cultured under standard conditions, were transfected with GFP and GFP-LNX1 expression constructs using calcium phosphate precipitation, and stable cell pools were selected using G418 antibiotic. To purify GFP-LNX1 and GFP protein complexes, ten confluent 15 cm dishes of cells were harvested and GFP affinity purifications performed using magnetic GFP-Trap^®^ beads, as per manufacturer’s instructions (ChromoTek). Purified complexes were separated by gel electrophoresis and stained using GelCode Blue Protein Stain (Thermo Scientific Pierce). Each lane was cut into slices for mass spectrometry analysis.

### Protein identification by mass spectrometry

Protein digestion, nano-liquid chromatography and MS/MS mass spectrometric analysis was performed at the Fingerprints Proteomics facility at University of Dundee, Scotland, UK. Proteins were identified by searching against the IPI protein database and data analysis was performed as previously described [8, 19]. Briefly, proteins identified in LNX complexes, but not in the control samples, were ranked according to Mascot protein scores and listed using protein symbols as identifiers. A Mascot protein score of 70 was then applied as a cut-off value to limit results to proteins that have been reliably identified, and probable environmental contaminants or false positives were eliminated as previously described [19].

### Characterisation of interactions by GFP pull-down assays

Expression vectors encoding GFP-tagged LNX1 or LNX2 constructs were transfected into HEK 293 cells, either alone or together with constructs encoding a LNX-interacting protein as previously described [8]. Briefly, cultures were harvested 24–48 hours post-transfection, and GFP affinity purification performed using 10ul GFP-Trap_M beads according to the manufacturer’s protocol. In some cases, the stringency of the wash conditions were increased by increasing the NaCl concentration in the standard wash buffer up to 500mM. Proteins were eluted by boiling in 2X SDS sample buffer and analysed by western blotting.

### Immunofluorescence staining

MCF7 cells (ATTC) were chosen for these experiments because of their flat morphology and good adherence to coverslips during the immunostaining procedure. They were grown under standard conditions on poly-D-lysine coated glass coverslips in a 6 well plate, were transfected with 2 μg of DNA using a calcium phosphate precipitation protocol. 24 hrs post transfection cells were washed with PBS and fixed with 4% PFA for 10 minutes at 4°C. Cells were incubated in blocking buffer (0.1% triton X-100, 5% goat serum and 2% bovine serum in PBS) prior to antibody incubation. All antibodies were diluted in 5% goat serum and 2% bovine serum in PBS (as described [20]). Cells were washed three times in PBS following each antibody incubation and coverslips were mounted onto glass slides using Fluoromount mounting media.

### Ubiquitination assays

Expression vectors encoding LNX proteins, LNX-interacting proteins and a HA epitope-tagged ubiquitin construct were co-transfected into HEK293 cells. Twenty hours after transfection, cells were incubated in fresh medium containing 10 μM MG132 (Merck Millipore). Cells were lysed in 100 μl 1% (w/v) Sodium Dodecyl Sulphate (SDS) supplemented with 15 mM *N*-Ethylmaleimide (NEM) and 1x Complete protease inhibitors (Roche Applied Sciences), and boiled for 5 min. Following cooling on ice, samples were diluted with 900 μl of ice cold buffer that contained 50 mM Tris (pH 8.0), 150 mM NaCl, 1% Triton, 0.5% sodium deoxycholate, 50 mM sodium fluoride, 0.1 mM sodium orthovanadate and protease inhibitors. Lysates were cleared by centrifugation at 13,000 rpm for 30 min at 4°C. Ubiquitinated proteins were immunoprecipitated overnight at 4°C from cell lysates using an anti-HA antibody. The ubiquitination status of substrate proteins was then revealed by Western blot analysis.

### Analysis of functional associations of LNX-interacting proteins

To compare interactions identified here with those from previous studies, lists of interacting proteins were taken from Wolting *et al*. [14], Guo *et al*. [15] and Lenihan *et al*. [8] and gene identifiers converted to gene symbols. These lists were combined and compared to the proteins identified here using Excel software (S2 Table). For analysis of functional associations, lists of gene symbols were submitted to the functional annotation tool in DAVID (Database for Annotation, Visualisation and Integrated Discovery; http://david.abcc.ncifcrf.gov/) using default settings [21].

### Statistical analysis

Two tailed Student *t* tests were performed using Microsoft Excel software. Where appropriate, data were analysed by one-way ANOVA, followed by Bonferroni post-hoc test using GraphPad Prism v.6.0 (La Jolla, CA, USA). *P* values of less than 0.05 were considered significant. Unless stated otherwise, all data are presented as mean ± SEM.

## Results

### Affinity purification and identification of LNX1 interacting proteins

The majority of LNX-interacting proteins have been identified by yeast-two hybrid assays and protein/peptide arrays [14, 15]. Only a minority of these have been confirmed in mammalian cells using full-length proteins that are targeted to their normal subcellular location. To directly identify interactions of LNX1 in a physiologically relevant context, we established stably-transfected HEK cells expressing GFP-tagged LNX1p80. We then affinity purified LNX1-containing protein complexes from these cells using GFP-Trap^®^ magnetic beads. Cells expressing GFP alone were used as a negative control for non-specific binding to either the beads or GFP tag. Purified proteins from both samples were identified by mass spectrometry. Non-specific interactions present in control GFP complexes, and likely false positives or environmental contaminants, were eliminated to generate a list of over 70 proteins specifically identified in affinity purified GFP-LNX1 complexes. The top 30 proteins ranked according to Mascot scores are shown in Table 1 and the full list is available as supplemental material online (S1 Table). The well-characterised LNX1-interacting proteins NUMB, NUMB-like and ERC2/CAST1 were specifically identified in GFP-LNX1 complexes–validating the overall approach. Examination of the carboxyl-terminal sequence of proteins that we identified indicates that many of them potentially contain PDZ binding motifs. Particularly noteworthy were proteins containing a putative PDZ-binding motif with a carboxyl-terminal cysteine.

**Table 1:** Proteomic analysis of GFP-LNX1 interacting proteins purified from HEK293 cells. The top 30 proteins identified, as ranked by Mascot score, are shown. The full table is available as supplementary material. Previously known interactions are underlined, as are carboxyl-terminal cysteine residues.

Gene Symbol	Mascot Score	Name	Carboxyl terminus
PPFIA1	5644	Isoform 1 of Liprin-alpha-1	DSATVRTYSC
LNX1	3662	Isoform 1 of E3 ubiquitin-protein ligase LNX	TIVSWPGTFL
MID2	2641	Isoform 1 of probable E3 ubiquitin-protein ligase MID2	PYVSGMKTCH
USP9X	2229	Isoform 2 of probable ubiquitin carboxyl-terminal hydrolase FAF-X	EVSPPQTKDQ
MYCBP2	1995	Probable E3 ubiquitin-protein ligase MYCBP2	CGVCRNAHTF
KIF7	1131	Kinesin-like protein KIF7	GMIDVRKNPL
KLHL11	1056	Kelch-like protein 11	RRVPSSQIEC
MID1	1030	Isoform 1 of Midline-1	DHLDCTEQLP
IARS	1020	Isoleucyl-tRNAsynthetase, cytoplasmic	VSVLPTTADF
PPFIA3	791	Isoform 1 of Liprin-alpha-3	DGVSVRTYSC
KIF14	629	Kinesin-like protein KIF14	ECTPSRIQWV
AKAP13	510	Isoform 6 of A-kinase anchor protein 13	VSAEGEEIFC
PEX1	494	Peroxisome biogenesis factor 1	FRPGQKVTLA
NUMB	438	Isoform 1 of Protein numb homolog	DLQKTFEIEL
RPL4	391	60S ribosomal protein L4	PTTEEKKPAA
NUMBL	356	Numb-like protein	DLQKTFEIEL
AP2M1	352	Isoform 1 of AP-2 complex subunit mu	GRSGIYETRC
PLEK	341	Pleckstrin	AIQMASRTGK
PPP1CA	294	Serine/threonine-protein phosphatase PP1-alpha catalytic subunit isoform 3	PPRNSAKAKK
TRIM27	279	Isoform Alpha of Zinc finger protein RFP	NHGHSMETSP
DUSP14	265	Dual specificity protein phosphatase 14	SRHLMPYWGI
TMED10	253	Transmembrane emp24 domain-containing protein 10	RFFKAKKLIE
ZNF24	248	Isoform 1 of Zinc finger protein 24	AEKLLNVVKV
ZCRB1	247	Zinc finger CCHC-type and RNA-binding motif-containing protein 1	YFSDEEELSD
AP2A1	246	Isoform B of AP-2 complex subunit alpha-1	HLCELLAQQF
LARS	244	Leucyl-tRNAsynthetase, cytoplasmic	IGDTIIYLVH
IQGAP1	242	RasGTPase-activating-like protein IQGAP1	FLLNKKFYGK
RPS27L	237	40S ribosomal protein S27-like	EGCSFRRKQH
CHD2	234	Isoform 2 of Chromodomain-helicase-DNA-binding protein 2	PDYNWNVRKT
ERC2	226	ERC protein 2	DQDDEEGIWA

### Confirmation and characterisation of LNX1 interactions

We chose five of the most reliably identified novel GFP-LNX1 interacting proteins from our proteomics study for further confirmation and analysis (liprinα1, MID2, KIF7, KLHL11 and AKAP13). The choice of these particular proteins was based on our ability to obtain cDNAs to make expression constructs or antibodies to detect them. We also examined two proteins (EphA7 and SynGAP1) that we previously demonstrated to interact with both LNX1 and LNX2 in yeast two-hybrid assays [13]. We first assessed the specificity of the interaction of these proteins with LNX1 and LNX2 by western blotting after “pull down” of GFP-tagged LNX proteins (Fig 1B). As expected from previous yeast two-hybrid data, SynGAP1 and EphA7 were able to bind both LNX1 and LNX2 in a GFP pull down assay, as was KLHL11. By contrast, liprin-α1, MID2, KIF7 and AKAP13 interacted specifically with LNX1 but not LNX2.

We then attempted to map these interactions to particular regions or individual domains of LNX1. We used amino-terminal (RING-NPAY motif) and carboxyl-terminal (PDZ1-PDZ4) fragments of LNX1, as well as constructs encoding the individual LNX1 PDZ domains, to map these interactions within LNX1 (Fig 1B). All the novel interactions mapped to the C-terminal PDZ domain region. As expected, NUMB was seen to bind the N-terminal fragment containing the NPAY motif, which serves as a positive control to show that this construct was fully functional in our assay. Interactions with individual PDZ domains were generally less prominent than with the full-length or PDZ1-PDZ4 constructs, despite equivalent amounts of GFP-tagged LNX constructs being expressed and pulled down in the assays. Nevertheless, the interactions of liprin-α1, KLHL11 and AKAP13 could be clearly mapped to LNX1 PDZ2, whereas KIF7 seemed to bind PDZ2 and PDZ4. SynGAP1 showed interactions with PDZ1, and to a somewhat lesser extent PDZ2, while the MID2 and EphA7 interactions could not be mapped clearly to any individual PDZ domain.

### Co-localisation between LNX and novel interacting proteins

**Fig 2:**
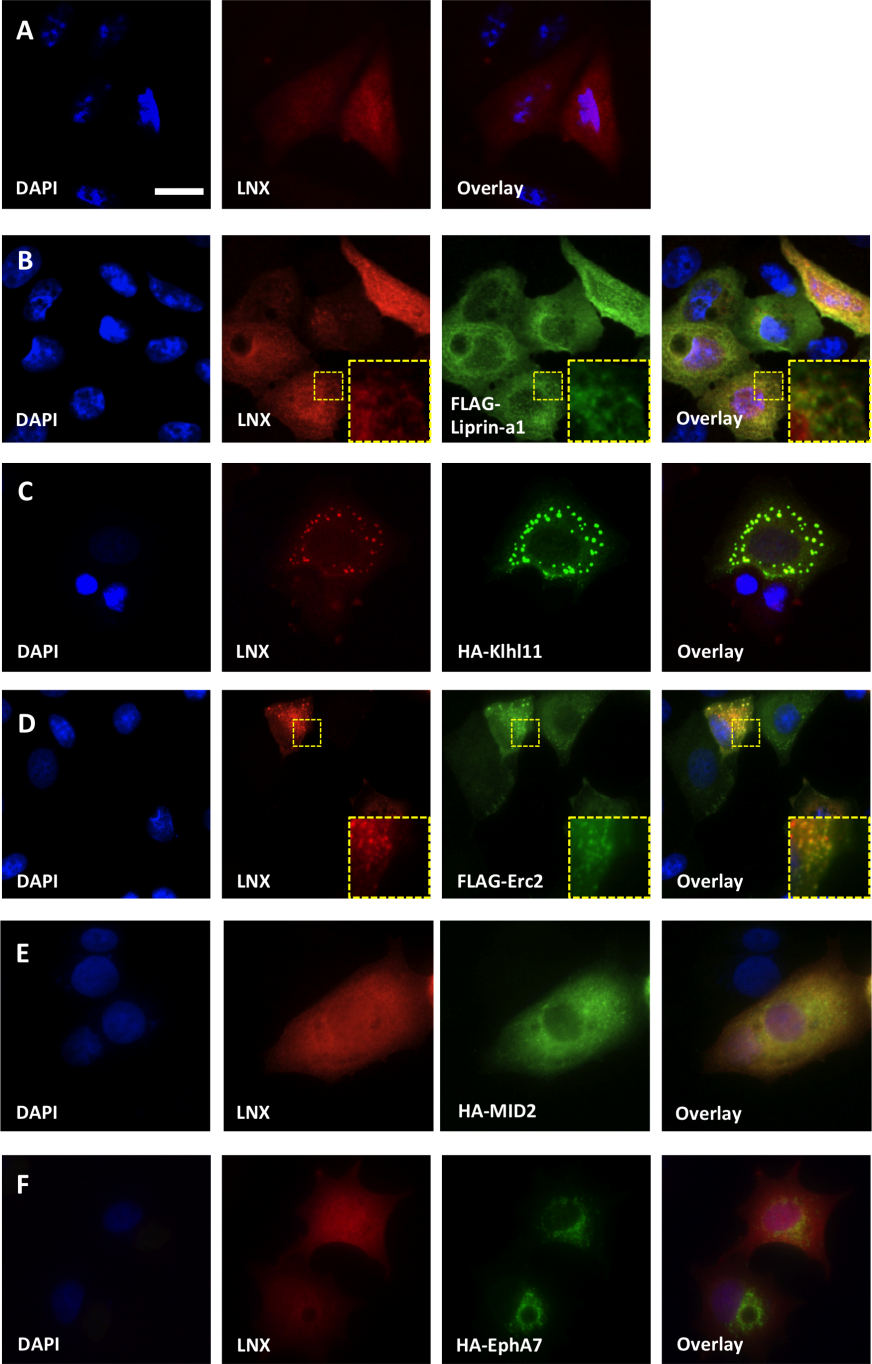
Representative fluorescence immunocytochemistry images examining localisation of the LNX1 and interacting proteins following transient expression in MCF-7 cells. **(A)** LNX1p80 transfected alone **(B)-(F)** LNX1p80 transfected in combination with the indicated interacting proteins that had been tagged with either HA or FLAG epitope tags. Anti-FLAG (green), anti-HA (green) and anti-LNX (red) were used to visualize the proteins of interest. Nuclei were stained with DAPI (blue). The different wavelengths were scanned individually and digitally merged (*overlay*). The regions highlighted by the small dashed boxes in B and D are shown enlarged in the bottom right corners of these images. Scale bar indicates 20 μm.

To further evaluate the relevance of these interactions we sought to assess potential co-localisation of LNX1 with some of these interacting proteins in a cellular context. LNX1p80 was co-expressed with either HA- or FLAG-epitope-tagged interacting proteins in MCF-7 cells and detected by immunofluorescence microscopy. In this analysis we observe partial co-localisation of liprin-α1 with LNX1 in the cytoplasm, particularly in structures in the perinuclear region of co-expressing cells (Fig 2B). This staining pattern is in contrast to the relatively diffuse localization of LNX1 in both the cytosol and nucleus when expressed alone (Fig 2A). KLHL11 shows a striking punctate cytoplasmic staining pattern and LNX1 redistributes to these structures when co-expressed with KLHL11, demonstrating clear co-localization (Fig 2C). Erc2 exhibits a diffuse cytoplasmic localization pattern but with some small punctate structures discernible in which co-staining for LNX1 is observed (Fig 2D). MID2 has a diffuse cytoplasmic localization similar to LNX1, but no clear co-localization in discrete structures was observed (Fig 2E). EphA7 localized exhibited a perinuclear staining which possibly represents localization of this transmembrane protein in the endoplasmic reticulum and/or Golgi, but LNX1 remained diffusely distributed in co-expressing cells and did not prominently co-localize with EphA7, suggesting that they do not interact extensively in this cellular context (Fig 2F). In summary then this analysis provides direct evidence for the co-localization of LNX1 with liprin-α1, KLHL11 and ERC2, while the diffuse cytoplasmic localization of both LNX1 and MID2 is at least compatible with their interaction in a cellular context.

### LNX1 binds the carboxyl-terminus of liprin-α1 and promotes its ubiquitination in both a RING domain dependent and independent manner

**Fig 3:**
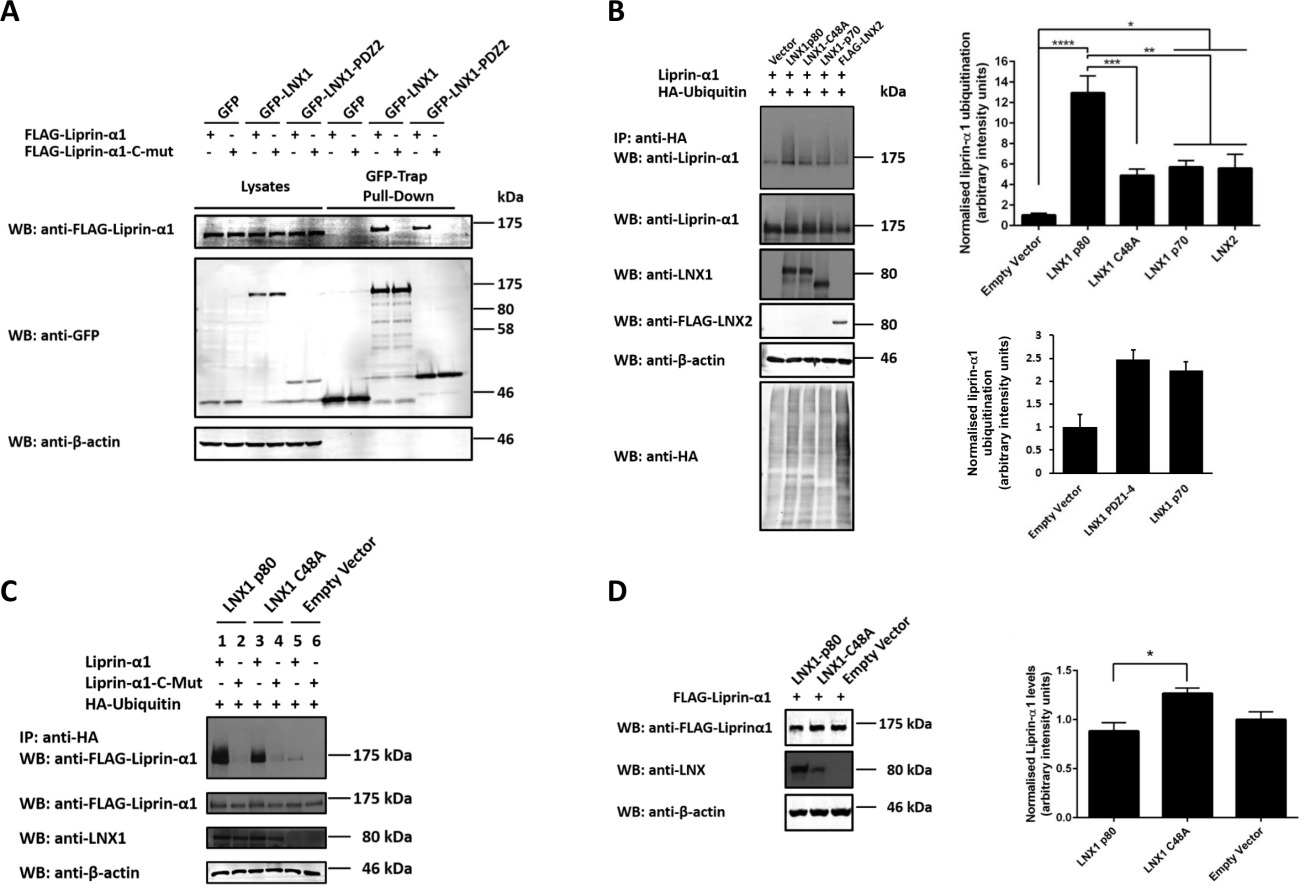
Liprin-α1 interacts with LNX1 via its C-terminus and is a substrate for LNX-mediated ubiquitination. **(A)** GFP pull-down assays performed on HEK 293T cells transiently transfected with either wild-type FLAG-liprin-α1 or a similar liprin-α1 construct with a carboxyl-terminal YSC* to DSE* mutation (FLAG-liprin-α1-C-mut) and either GFP-LNX1, GFP-LNX1-PDZ2 or GFP. Cell lysates and purified proteins were subjected to western blotting (WB) with the indicated antibodies. n = 2. **(B)** Ubiquitination of liprin-α1 assessed in a cell based assay. HEK 293T cells were co-transfected with the indicated LNX and liprin-α1 expression constructs as well with a construct encoding HA epitope-tagged ubiquitin. Ubiquitinated proteins were immunoprecipitated from cell lysates using an anti-HA antibody. Western blotting of immunoprecipitates for liprin- α1 revealed its ubiquitination as a high molecular weight smear. Western blot of whole cell lysates confirmed expression of all constructs. Levels of ubiquitinated liprin-α1 were quantified by densitometry and normalised for liprin-α1 expression in whole cell lysates. Data in the upper graph are expressed as mean ± SEM. n = 4. *p < 0.05, **p < 0.01, ***p < 0.001, ****p < 0.0001; one-way ANOVA followed by Bonferroni post-hoc test. Data from a separate experiment are shown in the lower graph as mean ± SEM. n = 3. **(C)** Ubiquitination of wild-type FLAG-liprin-α1 or FLAG-liprin-α1-C-mut in the presence of the indicated LNX1 constructs was assessed as in (B) above. An obvious high molecular weight smear corresponding to ubiquitinated wild-type liprin-α1, but not liprin-α1-C-mut with a carboxyl-terminal mutation, was detected for both wild-type LNX1p80 and the catalytically inactive LNXp80-C48A mutant. **(D)** Liprin-α1 protein levels assessed in the presence of either wild-type LNX1p80 or the LNXp80-C48A mutant. Whole cell lysates were analysed by Western blot using anti-FLAG, anti-LNX1/2-PDZ3/4 and anti-β-actin antibodies. Liprin-α1 protein levels were quantified, normalised to β-actin levels and subjected to one-way ANOVA followed by Bonferroni post-hoc test. Data are expressed as mean ± SEM. n = 4. *p < 0.05.

Many PDZ domains bind to carboxyl terminal motifs of their interaction partners, with the amino acids at positions 0 and -2 relative to the carboxyl terminus being particularly crucial for recognition by PDZ domains [22]. Liprin-α1 is one of several proteins in our proteomic analysis that contains a putative PDZ binding motif with a cysteine at its C-terminus (Table 1; S1 Table). To test if this motif was indeed required for binding to LNX1, a mutant of liprin-α1 was generated in which the carboxyl-terminal sequence YSC* was changed to DSE*. This mutation was found to abolish the binding of liprin-α1 to both full length LNX1 and also to LNX1 PDZ2, as assessed by GFP-Trap pull-down assays (Fig 3A). These results strongly indicate that liprin-α1 binds directly to the second PDZ domain of LNX1 through its carboxyl-terminal PDZ-binding motif.

We next assessed the ability of LNX proteins to ubiquitinate liprin-α1. For this purpose, liprin-α1, HA-tagged ubiquitin and various LNX proteins were co-expressed in HEK 293T cells in the presence of the proteasome inhibitor, MG132. Ubiquitinated proteins were specifically immunoprecipitated using an anti-HA antibody and detected by western blotting. Liprin-α1 ubiquitination was detected as a high molecular smear above the position of the main liprin-α1 band and quantified by densitometry analysis (Fig 3B). One-way ANOVA revealed significant differences in the ubiquitination status of liprin-α1 in the presence of LNX (*p* < 0.0001, F_4,15_ = 17.15). Ubiquitination in immunoprecipitates from cells expressing LNX1p80 was significantly stronger than in those from cells transfected with the control vector (*p* < 0.0001), as revealed by Bonferroni post-hoc test, indicating that LNX1p80 strongly promotes ubiquitination of liprin-α1. Surprisingly, cells expressing LNX1p80 containing the catalytically inactive RING-finger domain, (LNX1p80C48A) [4], also exhibited increased ubiquitination compared to the control vector, though not to the level observed for the wild type protein. This increase did not quite reach statistical significance but statistically significant increases in ubiquitination of liprin-α1 were observed in immunoprecipitates from cells transfected with the neuronal LNX1p70 isoform (*p* < 0.05), which lacks the RING finger domain required for ubiquitination, and LNX2 (*p* < 0.05), compared to the control vector (Fig 3B, upper graph). These increases in ubiquitination were of a similar magnitude to those seen for samples transfected with the catalytically inactive RING-finger domain mutant LNX1p80C48A. We next tested a LNX1 construct consisting of just the four PDZ domains and saw that this region of LNX1 was sufficient to promote liprin-α1 ubiquitination to a similar extent as LNX1p70 (Fig 3B, lower graph). These observations suggest that in addition to directly ubiquitinating liprin-α1 in a RING-domain dependent manner, LNX1 can promote liprin-α1 ubiquitination indirectly. Both of these effects are dependent on the LNX1: liprin-α1 interaction, since the ubiquitination of liprin-α1 induced by both LNX1p80 and LNX1p80C48A is abrogated when the liprin-α1 carboxyl terminal PDZ binding motif is mutated (Fig 3C).

We next assessed whether the ubiquitination of liprin-α1 induced by LNX1 promotes its degradation by the proteasome or other mechanisms. FLAG-liprin-α1, HA-ubiquitin and LNX1p80, LNX1p80C48A or vector only were co-transfected into HEK 293T cells. Quantitative western blot analysis of liprin-α1 in total cell lysates followed by one-way ANOVA indicated a significant effect of LNX on the levels of liprin-α1 in transfected cell lysates (*p* = 0.0134, F_2,9_ = 7.236; Fig 3D). Bonferroni post-hoc analysis, however, did not reveal a significant reduction of liprin-α1 in wild-type LNX1p80 or mutant LNX1p80C48A transfected cell lysates, compared to the control vector. Instead liprin levels were significantly increased when co-transfected with the ubiquitination deficient LNX1p80C48A mutant compared to the wild type LNX1p80. These results suggest that the strong ubiquitination of liprin-α1 by LNX1p80 wild type does not target liprin-α1 for proteasomal degradation and that LNX1p80C48A, despite also promoting a degree of ubiquitination, has a slight stabilizing effect on liprin-α1 levels.

### KLHL11, KIF7 and ERC2 are substrates for LNX-mediated ubiquitination substrates

**Fig 4:**
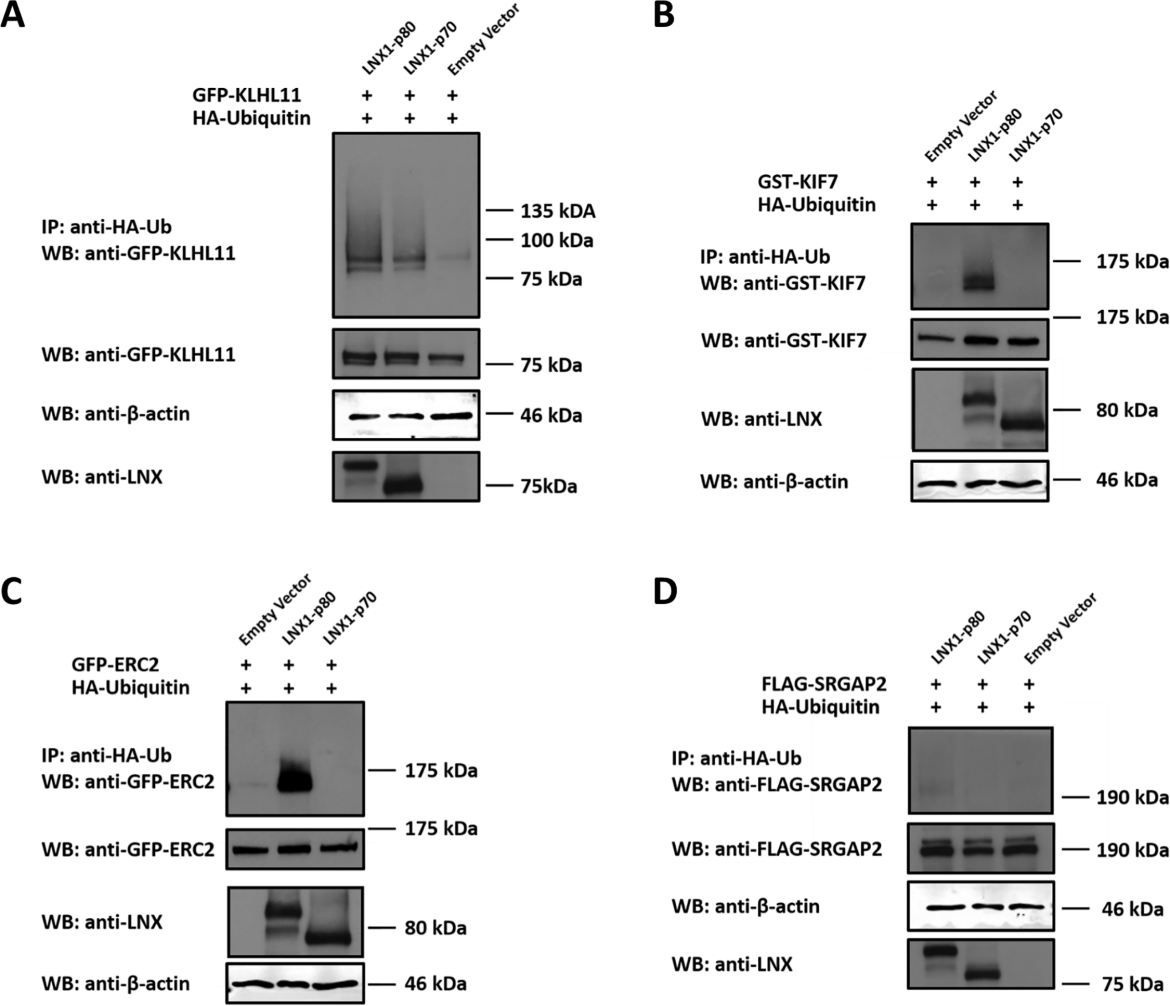
LNX1 mediated ubiquitination of KLHL11, KIF7 and ERC2. HEK 293T cells were transiently transfected with the indicated constructs and ubiquitinated proteins detected as in Fig 3B above. The ubiquitination status of **(A)** KLHL11, **(B)** KIF7, **(C)** ERC2 and **(D)** SRGAP2 was then revealed by Western blot analysis using the indicated antibodies. Western blot of whole cell lysates confirmed expression of all constructs. n = 2.

Having shown that liprin-α1 is ubiquitinated by LNX, we next investigated whether a number of the other LNX1 interacting proteins identified were also LNX1 substrates in similar cell-based ubiquitination assays. HEK 293T cells were transiently transfected with GFP-KLHL11, GST-KIF7, GFP-ERC2, or FLAG-SRGAP2, HA-Ubiquitin and LNX1p80, LNX1p70 or vector only. LNX1p80 increased ubiquitination of KLHL11, KIF-7, ERC2 and possibly SRGAP2 to a much lesser extent (Fig 4A, 4B, 4C and 4D respectively). As was the case for liprin-α1, expression of LNX1p70 increased ubiquitination of KLHL11 (Fig 4A), but appeared to have no effect on KIF7, ERC2 or SRGAP2 (Fig 4B, 4C and 4D respectively). These results underline the ability of LNX1p80 to ubiquitinate its interacting proteins via its p80 RING domain, but also further support a RING domain-independent function for LNX1p70 in enhancing ubiquitination of certain ligand proteins.

### Interaction of LNX1 with members of the TRIM E3 ubiquitin ligase family

**Fig 5:**
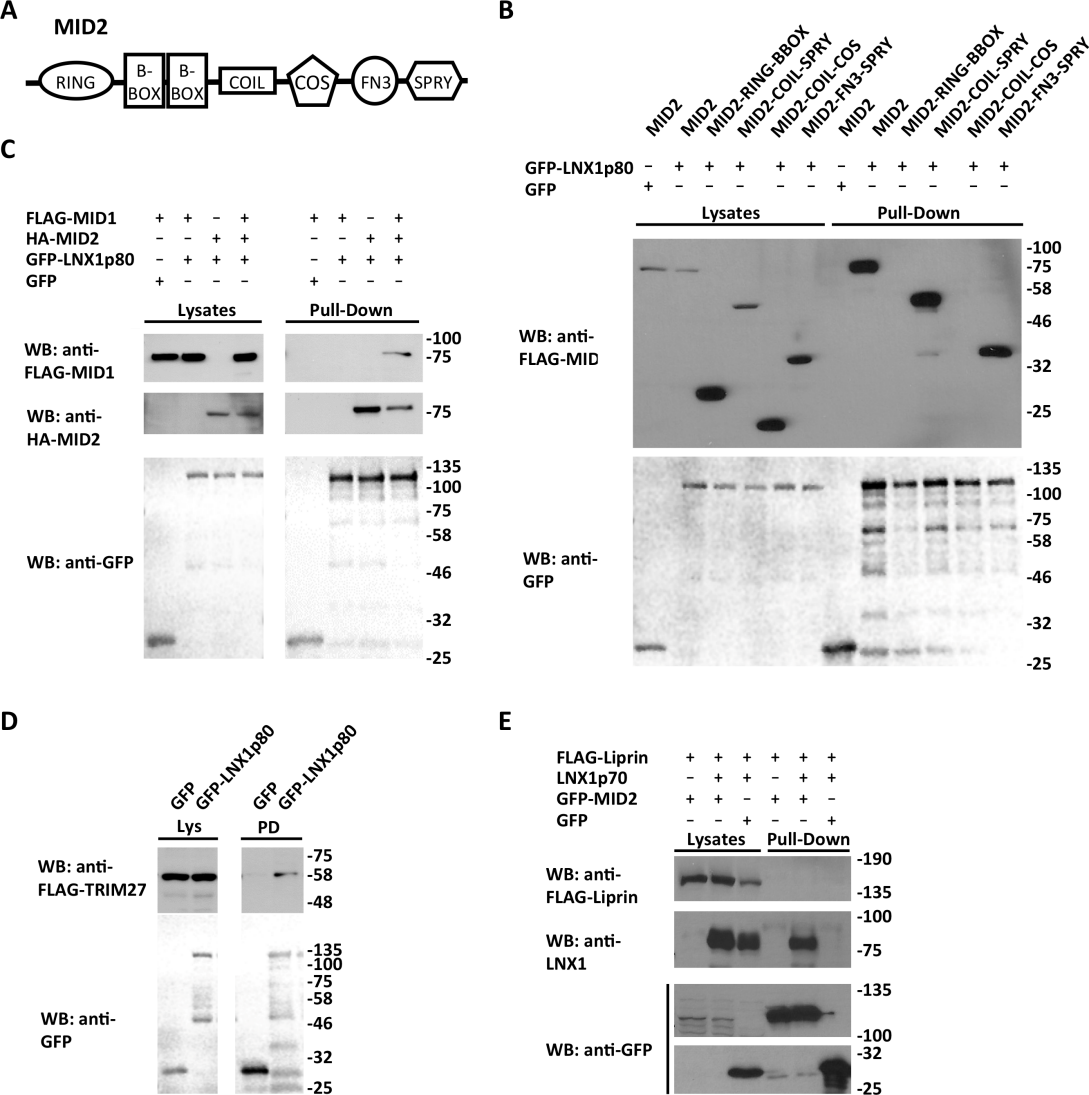
Interactions of LNX1 with the TRIM E3 ubiquitin ligase family. A, Schematic diagram of the domain structure of MID2 showing the RING and B-BOX type zinc finger domains, the coiled-coil dimerization domain (COIL), the microtubule-binding COS (C-terminal subgroup one signature) domain, the fibronectin type III (FN3) domain and the SPRY (in splA kinase and ryanodine receptor) domain. MID1 shares a very similar domain organization, whereas TRIM27 lacks the COS and FN3 domains [24]. B, Mapping of the LNX1 binding site on MID2. The ability of the indicated FLAG epitope tagged MID2 constructs to interact with GFP-tagged LNX1p80 was assessed following transfection into in HEK 293 cells by GFP “pull down” assays. Successful expression of constructs was verified by western blotting of cell lysates and interactions detected in pull down samples. n = 2. C, Analysis of LNX1 binding to MID1. The ability of FLAG epitope-tagged MID1 to interact with GFP-tagged LNX1p80 was assessed in the presence or absence of HA epitope-tagged MID2. n = 2 D, Confirmation of TRIM27 interaction with LNX1 in a GFP “pull down” assay. n = 2. E, Investigation of the ability of MID2, LNX1p70 and liprin-α1 to form a trimolecular complex. Interaction of untagged LNX1p70 and FLAG-tagged liprin-α1 with GFP-tagged MID2 was assessed in GFP “pull down” assays. n = 3.

We hypothesized that LNXp70 might act as a scaffold to recruit other E3 ubiquitin ligases to substrates that bind to its PDZ domains. Since six E3 ubiquitin ligases (MID1, MID2, MycBP2, TRIM27, TRAF4 and DZIP3) were identified in the LNX1 interactome we sought to further characterize some of these interactions (Table 1). We focused on the three members of the tripartite motif (TRIM) family identified in our mass spectrometry data—MID1/TRIM18, MID2/TRIM1 and TRIM27. We had already seen that transfected, epitope-tagged MID2 interacted with the PDZ domain region of LNX1, but that this interaction could not be mapped to an individual PDZ domain (Fig 1B). Furthermore the histidine residue present at the carboxyl terminus of MID2 (Table 1) does not fit with consensus sequences for PDZ binding motifs [15, 23]. These observations suggest that MID2 does not bind LNX via a typical carboxyl terminal: PDZ domain interaction. We therefore investigated which part of MID2 mediates this interaction (Fig 5A and 5B). We found that a region containing the FN3 and SPRY domains of MID2 is necessary and sufficient to bind LNX1, with no interaction being observed for constructs containing the RING, B-BOX, coiled-coil or microtubule binding COS domains.

We next sought to confirm the interaction of LNX1 with MID1 and TRIM27. Surprisingly, no interaction with LNX1p80 was seen for transfected epitope-tagged MID1 under the same experimental conditions for which MID2 binding was observed (Fig 5C). However, when MID1 and MID2 were co-transfected with LNX1, MID1 did co-purify with LNX1, suggesting that MID1 interacts indirectly with LNX1 because of its ability to heterdimerize with MID2 via the coiled coil regions of both proteins [25]. By contrast, we were able to observe the interaction of transfected epitope-tagged TRIM27 with LNX1 without having to co-transfect MID2, confirming this interaction and suggesting that it is direct rather than mediated by heterodimerization with MID2 (Fig 5D).

Given the robust interaction of MID2 with the LNX1 PDZ domains we sought to test the idea that the LNX1p70 isoform, while lacking its own RING domain, might target the ubiquitin ligase activity of the MID2 RING domain to proteins that bind the LNX1 PDZ domains. To do this we examined whether MID2, LNX1p70 and the LNX1 ligand liprin-α1 could form a ternary complex (Fig 5D). While LNX1p70 was seen to interact strongly with GFP-tagged MID2, we could not observe co-purification of liprin-α1 with LNX1p70 and MID2. It is thus unlikely that the recruitment of MID2 E3 ligase activity by LNX1p70 could explain the ability of LNX1p70 to promote liprin-α1 ubiquitination. However, confirmation of the ability of LNX1p70 to bind strongly to MID2 indicates that LNX1p70 might recruit MID2 to mediate ubiquitination of other LNX1 interacting proteins.

## Discussion

In an analysis of the human PDZ domain-ligand interactions network (PDZNet), LNX1 ranked third in terms of total number of interactions [26]. The large number of interactions that have been reported for LNX1 is remarkable, given that it is not a well-studied protein. Considering that LNX protein levels are tightly regulated [5, 8, 18], establishing which LNX interactions are physiologically relevant is a key challenge. To complement previous approaches, we have carried out the first affinity purification/mass spectrometry-based analysis of the LNX1 interactome in the context of mammalian cells. Our results provide confirmation of just 10 of the approximately 400 previously reported LNX interactions [8, 14, 15] (S2 Table). The identification of well-characterised interaction partners, such as NUMB and ERC2, validates our methodology, and supports the veracity of these interactions. The failure to confirm more previously reported interactions may simply be because these proteins are not expressed in HEK cells. However, this seems unlikely to be true in all cases, and it may be that some previously reported LNX binding protein were not detected in our study because, (1) they do not interact with full-length LNX proteins, (2) they are localised in a different subcellular compartment from LNX, or (3) they are out-competed by ligands present in HEK cell lysates that have a higher affinity. Thus, some interaction pairs that have been identified by yeast-two-hybrid or protein/peptide arrays may not be significant in a mammalian cellular context in which many potential ligands are binding competitively to LNX proteins. In addition, indirect interactions detected by our affinity purification approach may partly explain the lack of overlap with previously identified LNX ligands. For example, KIF7 lacks a strong carboxyl terminal PDZ-binding consensus sequence, but is known to interact with liprin-α1 [27], suggesting that the interaction of KIF7 with LNX1 could be mediated by liprin-α1. Overall though, our data confirm the propensity of LNX proteins to interact with a large number of ligands and may highlight those interactions that are physiologically relevant.

Our goal in characterising the LNX1 interactome was to gain insights into potential functions of LNX proteins and to identify differential functions of LNX1 and LNX2. We used the DAVID Bioinformatics Resource [21] to identify significant biological associations of proteins present in LNX1 complexes. Functional annotation terms that are enriched in the LNX1 interactome include: ATP/nucleotide binding, microtubule cytoskeleton/microtubule-based process, translation/protein biosynthesis, ER-Golgi intermediate compartment, protein transport/localisation, peroxisome membrane, protein kinases, protein phosphatases, cell projection morphogenesis/organisation, synaptosome, regulation of synaptic transmission, ubiquitin conjugation pathway, cell division, zinc/RING finger domain-containing and emp24 domain-containing. Examination of the proteins identified, also reveals several incidences of multiple members of a protein family being identified. These families (with family members identified in parentheses) include: NUMB (NUMB, NUMBL), Midline probable E3 ligases (MID1, MID2), peroxisome biogenesis/assembly factors (PEX1, PEX6), tRNA synthetases (IARS, LARS, MARS), ELKS/Rab6-interacting/CAST family (ERC1, ERC2), Flotillin (FLOT1, FLOT2), Kinesin-like proteins (KIF7, KIF14), liprin-α proteins (PPFIA1, PPFIA3) and Transmembrane emp24 domain-containing proteins (TMED2, TMED4, TMED5, TMED9, TMED10). While the liprin, NUMB and ERC family members have conserved carboxyl-terminal PDZ-binding consensus motifs, the other families do not have conserved carboxyl-termini that would explain their interaction with LNX PDZ domains. Nevertheless, these novel associations represent valuable clues regarding putative functions of LNX1 and merit further investigation.

The *Lnx1* and *Lnx2* genes arose by gene duplication early in the vertebrate lineage [13]. The invertebrate *Lnx1/2-like* gene is more similar to *Lnx2*, indicating that *Lnx1* may have may have undergone neofunctionalisation following the gene duplication[13]. We identified several LNX1-specific interactions (liprin-α1, MID2, AKAP13 and KIF7) that could mediate functions that are unique to LNX1. Liprin-α1 and AKAP13 have putative PDZ binding motifs with a carboxyl-terminal cysteine, and interact specifically with LNX1 via PDZ2, indicating that LNX1-PDZ2 seems to have a preference for ligands with carboxyl-terminal cysteines compared to LNX2-PDZ2. This would agree with previous reports [8, 15]. The molecular basis for the specificity of these interactions with LNX1 versus LNX2 is not obvious however, as key residues involved in ligand recognition are conserved between the PDZ2 domains of LNX1 and LNX2 [13] and some interacting proteins with a carboxyl-terminal cysteine such as KLHL11, seem to interact equally well with both LNX1 and LNX2.

We have characterised the interaction with liprin-α1 in more detail by verifying that the interaction is dependent on the carboxyl-terminal–YSC* motif and showing that liprin-α1 is a substrate for ubiquitination by LNX1p80. However, ubiquitination of liprin-α1 by LNX1 did not significantly alter liprin-α1 protein levels. This suggests that LNX1-mediated ubiquitination does not target liprin-α1 for proteasomal (or lysosomal) degradation, but perhaps affects some other aspect of liprin-α1 function. While liprin-α1 is widely expressed in many tissues, liprin-αs are best characterised as a component of the presynaptic cytomatrix of the active zone (CAZ) complex, that is involved in synapse maturation in neurons [28]. While LNX1p80 could ubiquitinate liprin-α1 in non-neuronal tissues, neurons are thought to exclusively express the LNX1p70 and LNX1p62 isoforms that lack the catalytic RING domain. Surprisingly, we found that LNX1p70 was able to promote ubiquitination of liprin-α1, albeit to a lesser extent than LNX1p80. A similar effect was observed for ubiquitination of KLHL11.

These findings suggest that the neuronal LNX1p70 isoform may be able to recruit other E3 ligases to mediate ubiquitination of substrates that bind to its PDZ domains. Notably we identified six E3-ubiquitin ligases in our LNX1 interactome (MID1, MID2, MYCBP2, TRIM27, TRAF4 and DZIP3) and some were identified in previous studies [29]. MID1/TRIM18, MID2/TRIM1 and TRIM27 are members of the large TRIM family of E3-ubiquitin ligases [24]. We could confirm the interaction of MID2 and TRIM27 with LNX1, while MID1 seemed to require the co-expression of MID2 to interact with LNX1, suggesting its interaction is indirect and mediated by its known ability to heterodimerize with MID2 [25]. The MID2 interaction mapped to the PDZ region of LNX1, though not clearly to any one PDZ domain, while the FN3 and SPRY domains of MID2 are required for the interaction. The carboxyl terminal sequences of MID2 and TRIM27 are not closely related and don’t match consensus PDZ binding motifs (Table 1), arguing against a canonical carboxyl terminal: PDZ domain mode of binding. Since TRIM27 lacks an FN3 domain it may be the SPRY that mediates the interaction of LNX with MID2 and TRIM27. However there is evidence in the IntAct database (http://www.ebi.ac.uk/intact/) that MID2 can dimerize with TRIM27, and so we cannot completely rule out the possibility that the interaction of TRIM27 with LNX1 might be mediated by endogenous MID2 in our experiments.

To explore whether MID2 might be responsible for the ability of LNX1p70 to promote ubiquitination of liprin-α1 we tested the ability of these three proteins to form a ternary complex. Robust binding of LNX1p70 to GFP-tagged MID2 was observed, but co-purification of liprin-α1 was not observed in this experimental setup. It may be that binding of MID2 and liprin-α1 to LNX1 is competitive, or that the formation of a ternary complex is very transient. While we were not able to obtain direct evidence for LNX1p70 acting to scaffold an interaction of MID2 with liprin-α1 in this case, it will be interesting to test this hypothesis for other combinations of LNX1-interacting E3 ligases and substrates including TRIM27 and KLHL11. Such a mechanism, if proven, could explain the existence and conservation of the LNX1p70 isoform in diverse vertebrate species despite its lack of catalytic activity [13].

Another component of the presynaptic CAZ complex that we identified is the known LNX1 interacting protein ERC2/CAST1. ERC2 was previously shown to bind to LNX1-PDZ2 via a carboxyl terminal IWA* motif and to co-localise with LNX1 in neurons [30]. We show here for the first time that LNX1p80 but not LNX1p70 causes ubiquitination of ERC2. However this may not be so relevant *in vivo* since ERC2 (and the isoform of ERC1 that has the IWA* motif) are exclusively expressed in the brain [31], whereas LNX1p80 is expressed in non-neuronal tissues [32]. Instead LNX2, which can also interact with ERC1 and ERC2 [8], is perhaps more likely to promote their ubiquitination in neurons *in vivo*. Mice lacking LNX2 as well as the neuronal LNX1p70 and p62 isoforms have recently been shown to exhibit decreased anxiety-related behaviour [8]. Notably these mice don’t show observable differences in levels of NUMB proteins. The ability of LNX proteins to bind to, and potentially promote ubiquitination of the prominent presynaptic CAZ complex components liprin-α1, liprin-α3, ERC1 and ERC2 provides a putative mechanism whereby loss of LNX proteins might cause an anxiety related phenotype through altered synaptic function. In addition, we have identified many other proteins with well-established functions in neuronal development and synapse formation in the LNX1 interactome. For example, KIF7, a kinesin motor protein involved in Hedgehog signalling, and MID2, an E3 ubiquitin ligase, both function in neural development [33, 34], as does SRGAP2[35], while MYCBP2 (Pam/highwire/rpm-1) is a well-known regulator of synapse formation. Overall, the LNX1 interacting proteins and substrates identified and characterised here are plausible candidates that may, in addition to NUMB, mediate physiological functions of LNX proteins in the CNS as well as other tissues.

## Supporting information

S1 Table Proteomic analysis of GFP-LNX1 interacting proteins purified from HEK293 cells. (DOC)

S2 Table Comparison of LNX1-interacting proteins identified here with those identified in previous studies. (XLSX)

## Abbreviations

PDZ: PSD-95, DlgA, ZO-1
RING: Really Interesting New Gene
LNX: Ligand of NUMB protein X
CNS: Central Nervous System
ERC: ELKS/Rab6-interacting/CAST
SPRY: (in splA kinase and ryanodine receptor)
TRIM: tripartite motif
FN3: fibronectin type III
COS: C-terminal subgroup one signature
