# Supplementary material for: Proteomic analysis reveals novel ligands and substrates for LNX1 E3 ubiquitin ligase
Source: PLoS One. 2017 Nov 9;12(11):e0187352. doi: 10.1371/journal.pone.0187352 (PMC5679597; doi:10.1371/journal.pone.0187352)
Supplement: S1 Table — (DOC) [file pone.0187352.s001.doc]

**S1 Table. Proteomic analysis of GFP-LNX1 interacting proteins purified from HEK293 cells** Previously known interactions are underlined, as are carboxyl-terminal cysteines.

| **Gene Symbol** | **Mascot Score** | **Name** | **Carboxyl terminus** |
| --- | --- | --- | --- |
| PPFIA1 | 5644 | Isoform 1 of Liprin-alpha-1 | DSATVRTYSC |
| LNX1 | 3662 | Isoform 1 of E3 ubiquitin-protein ligase LNX | TIVSWPGTFL |
| MID2 | 2641 | Isoform 1 of probable E3 ubiquitin-protein ligase MID2 | PYVSGMKTCH |
| USP9X | 2229 | Isoform 2 of probable ubiquitin carboxyl-terminal hydrolase FAF-X | EVSPPQTKDQ |
| MYCBP2 | 1995 | Probable E3 ubiquitin-protein ligase MYCBP2 | CGVCRNAHTF |
| KIF7 | 1131 | Kinesin-like protein KIF7 | GMIDVRKNPL |
| KLHL11 | 1056 | Kelch-like protein 11 | RRVPSSQIEC |
| MID1 | 1030 | Isoform 1 of Midline-1 | DHLDCTEQLP |
| IARS | 1020 | Isoleucyl-tRNAsynthetase, cytoplasmic | VSVLPTTADF |
| PPFIA3 | 791 | Isoform 1 of Liprin-alpha-3 | DGVSVRTYSC |
| KIF14 | 629 | Kinesin-like protein KIF14 | ECTPSRIQWV |
| AKAP13 | 510 | Isoform 6 of A-kinase anchor protein 13 | VSAEGEEIFC |
| PEX1 | 494 | Peroxisome biogenesis factor 1 | FRPGQKVTLA |
| NUMB | 438 | Isoform 1 of Protein numb homolog | DLQKTFEIEL |
| RPL4 | 391 | 60S ribosomal protein L4 | PTTEEKKPAA |
| NUMBL | 356 | Numb-like protein | DLQKTFEIEL |
| AP2M1 | 352 | Isoform 1 of AP-2 complex subunit mu | GRSGIYETRC |
| PLEK | 341 | Pleckstrin | AIQMASRTGK |
| PPP1CA | 294 | Serine/threonine-protein phosphatase PP1-alpha catalytic subunit isoform 3 | PPRNSAKAKK |
| TRIM27 | 279 | Isoform Alpha of Zinc finger protein RFP | NHGHSMETSP |
| DUSP14 | 265 | Dual specificity protein phosphatase 14 | SRHLMPYWGI |
| TMED10 | 253 | Transmembrane emp24 domain-containing protein 10 | RFFKAKKLIE |
| ZNF24 | 248 | Isoform 1 of Zinc finger protein 24 | AEKLLNVVKV |
| ZCRB1 | 247 | Zinc finger CCHC-type and RNA-binding motif-containing protein 1 | YFSDEEELSD |
| AP2A1 | 246 | Isoform B of AP-2 complex subunit alpha-1 | HLCELLAQQF |
| LARS | 244 | Leucyl-tRNAsynthetase, cytoplasmic | IGDTIIYLVH |
| IQGAP1 | 242 | RasGTPase-activating-like protein IQGAP1 | FLLNKKFYGK |
| RPS27L | 237 | 40S ribosomal protein S27-like | EGCSFRRKQH |
| CHD2 | 234 | Isoform 2 of Chromodomain-helicase-DNA-binding protein 2 | PDYNWNVRKT |
| ERC2 | 226 | ERC protein 2 | DQDDEEGIWA |
| PPP2R1A | 224 | Serine/threonine-protein phosphatase 2A 65 kDa regulatory subunit A alpha isoform | QEALTVLSLA |
| CSNK1D | 199 | CSNK1D Isoform 1 of Casein kinase I isoform delta | SSGLQSVVHR |
| PEX6 | 199 | PEX6 Peroxisome assembly factor 2 | KRIQRKFAAC |
| TRAF4 | 185 | TRAF4 Isoform 1 of TNF receptor-associated factor 4 | AVELPRKILS |
| MED7 | 180 | MED7 Mediator of RNA polymerase II transcription subunit 7 | VLIDEMNERP |
| NDEL1 | 177 | NDEL1 nuclear distribution protein nudE-like 1 isoform A | VIFPTLFMGQ |
| FBXO11 | 176 | FBXO11 F-box only protein 11 | PIESNTLQHN |
| TCEB1 | 175 | TCEB1 Transcription elongation factor B polypeptide 1 | LLMAANFLDC |

Supplemental Table 1 continued

| **Gene Symbol** | **Mascot Score** | **Name** | **Carboxyl terminus** |
| --- | --- | --- | --- |
| NMNAT1 | 160 | NMNAT1 Nicotinamide mononucleotide adenylyltransferase 1 | LQRNTAEAKT |
| AGMAT | 157 | AGMAT Agmatinase, mitochondrial | LCALPKVTTV |
| PPP2CA | 155 | PPP2CA Serine/threonine-protein phosphatase 2A catalytic subunit alpha isoform | VTRRTPDYFL |
| MRPL4 | 153 | MRPL4 Isoform 1 of 39S ribosomal protein L4, mitochondrial | QGPAATPYHC |
| C3orf26 | 149 | C3orf26 Uncharacterized protein C3orf26 | KSESLKLGLF |
| COPA | 148 | COPA Isoform 1 of Coatomer subunit alpha | GLRISPLQFR |
| LMAN2 | 145 | LMAN2 Vesicular integral-membrane protein VIP36 | KRQERNKRFY |
| PRKDC | 143 | PRKDC Isoform 1 of DNA-dependent protein kinase catalytic subunit | RTWEGWEPWM |
| EIF3H | 135 | EIF3H cDNA FLJ35809, clone TESTI2006016, highly similar to Eukaryotic translation initiation factor 3 subunit 3 | MAQALQEYNN |
| ERC1 | 135 | ERC1 Isoform 2 of ELKS/Rab6-interacting/CAST family member 1 | DQDEEEGIWA |
| MYH9 | 134 | MYH9 Isoform 1 of Myosin-9 | ADGAEAKPAE |
| DYNLRB1 | 132 | DYNLRB1 Isoform 1 of Dynein light chain roadblock-type 1 | FLIVIQNPTE |
| IGLV2-14 | 132 | IGLV2-14;IGLC2 IGL@ protein | CALWYSTHFV |
| PKP1 | 130 | PKP1 Isoform 2 of Plakophilin-1 | NSLRNFTSRF |
| CLK4 | 129 | CLK4 Dual specificity protein kinase CLK4 | HPFFDLLKKK |
| RPL36AL | 129 | RPL36AL 60S ribosomal protein L36a-like | KKRKGQVIQF |
| ZNF192 | 128 | ZNF192 Zinc finger protein 192 | SGEKSESISV |
| SERPINB3 | 124 | SERPINB3 Isoform 1 of Serpin B3 | ILFYGRFSSP |
| DNAJC9 | 123 | DNAJC9 DnaJ homolog subfamily C member 9 | KKSALKKEKK |
| GTF2E2 | 122 | GTF2E2 Transcription initiation factor IIE subunit beta | KDYSDITSSK |
| DZIP3 | 120 | DZIP3 Isoform 1 of E3 ubiquitin-protein ligase DZIP3 | GHPSRQLPKI |
| FLOT2 | 120 | FLOT2 Flotillin-2 | LIKKATGVQV |
| GRWD1 | 110 | GRWD1 Glutamate-rich WD repeat-containing protein 1 | GFTIFRTISV |
| KPNA1 | 109 | KPNA1 Importin subunit alpha-1 | CEAPMEGFQL |
| TMED2 | 109 | TMED2 Transmembrane emp24 domain-containing protein 2 | KRFFEVRRVV |
| PIGR | 108 | PIGR Polymeric immunoglobulin receptor | AEAQDGPQEA |
| CIT | 106 | CIT Isoform 1 of Citron Rho-interacting kinase | VNKVWDQSSV |
| SEC22B | 104 | SEC22B Vesicle-trafficking protein SEC22b | LIVYVRFWWL |
| TMED9 | 103 | TMED9 Transmembrane emp24 domain-containing protein 9 | KSFFEAKKLV |
| RBM12B | 96 | RBM12B Uncharacterized protein | GPRKVKLTLL |
| FLOT1 | 94 | FLOT1 Flotillin-1 | QVNHKPLRTA |
| MARS | 94 | MARS Methionyl-tRNAsynthetase, cytoplasmic | PEAPKGKKKK |
| CDK13 | 91 | CDK13 Isoform 2 of Cyclin-dependent kinase 13 | GRGRGRGLPY |
| PCM1 | 91 | PCM1 Isoform 1 of Pericentriolar material 1 protein | EPETVGAQSI |
| SMC4 | 86 | SMC4 Isoform 2 of Structural maintenance of chromosomes protein 4 | PKEIASKGLC |
| IKBKAP | 82 | IKBKAP Elongator complex protein 1 | RTQWKLSLLD |
| TMED5 | 76 | TMED5 Transmembrane emp24 domain-containing protein 5 | LFEDKRKSRT |
| TMED4 | 71 | TMED4 Isoform 1 of Transmembrane emp24 domain-containing protein 4 | KSFFEAKKLV |
